# Job dissatisfaction as a predictor of poor health among middle-aged workers: a 14-wave mixed model analysis in Japan

**DOI:** 10.5271/sjweh.3985

**Published:** 2021-10-31

**Authors:** Takashi Oshio

**Affiliations:** 1 Takashi Oshio, PhD, Institute of Economic Research, Hitotsubashi University, Tokyo, Japan

**Keywords:** job satisfaction, psychological distress, self-rated health

## Abstract

**Objective:**

This study aimed to examine the association between job dissatisfaction (JD) and health outcomes among middle-aged workers.

**Methods:**

This study used longitudinal data comprising 156 823 observations of 24 056 workers (13 177 men and 10 879 women) collected from a 14-wave nationwide population-based survey in Japan that began in 2005, involving individuals aged 50–59 years. Mixed models were estimated to examine the association between JD and the risk of psychological distress (PD), poor self-rated health (SRH), and health-related resignation (HRR).

**Results:**

Across all waves, 20.9–32.5% of participants were dissatisfied with their jobs for at least one year before each wave. Mixed model results showed that this JD experience was associated with higher risks of PD, poor SRH, and HRR, with odds ratios (OR) of 1.96 [95% confidence interval (CI) 1.75–2.20], 1.33 (95% CI 1.26 –1.40), and 1.57 (95% CI 1.40 –1.75), respectively. A longer JD duration was associated with a higher risk of poor health. No substantial differences between genders were found regarding the association between JD and health outcomes. A separate analysis showed reverse causation from poor health to JD; poor health was significant in predicting later JD even when it was controlled for.

**Conclusions:**

The results confirm that JD was predictive of poor health among middle-aged workers. Therefore, policymakers and managers should monitor the JD of their employees and improve their work environments to enhance their occupational health.

Job dissatisfaction (JD) is widely known to have a negative association with workers’ health and productivity (1–3). Many studies have observed that JD contributes to mental health problems, including anxiety, burnout, and depression (4–7), as well as poor self-rated health (SRH) (8–10), which represent more general health conditions. Studies have also provided evidence of a correlation between JD and more objective negative outcomes in the workplace, such as sickness absence (11–13), health-related job loss (14), and disability pension (15, 16), all of which signify a loss of effective workforce participation. JD can be interpreted as a key mediator of the association between psychosocial factors in the workplace and workers’ health outcomes (6, 7) because it reflects a worker’s subjective assessment of various aspects of the work environment.

However, the observed association between JD and health outcomes is most likely confounded by personality traits (17, 18) and other inherent individual attributes that are likely to vary from participant to participant. For instance, workers with higher neuroticism – that is, those who tend to respond to threats, frustration, or loss with negative emotions – are more likely to be dissatisfied with their jobs and suffer from psychological distress (PD) (17). If this is the case, the observed association between JD and health outcomes may be overstated. Many previous studies that relied on cross-sectional analysis were not fully free from this type of bias.

Another issue to be addressed is simultaneity bias. Health outcomes are affected by JD; however, JD is also likely to be affected by health outcomes, possibly resulting in an overestimated impact of JD on health, as observed in the cross-sectional data (19, 20). Prospective cohort studies are generally expected to alleviate these biases, but follow-up for just a few years after the baseline cannot fully control for the correlations between repeated measurements within each participant.

Lastly, the reverse causation from health outcomes to JD has been largely understudied. Although many studies have investigated the determinants of JD (21–26), health outcomes have usually been treated as a result rather than a cause of JD.

In this study, we examined the association between JD and health among middle-aged workers using longitudinal data obtained from a 14-wave nationwide population-based survey. Unlike most preceding studies, we employed a mixed model analysis (27) to take full advantage of repeated measures for each participant over time. Mixed models can capture both fixed effects (which are assumed to remain the same across participants) and random effects (which are assumed to vary randomly from participant to participant). These models are expected to control for participant-specific factors, which may confound the association between JD and health outcomes by explicitly accounting for the correlations between repeated measurements within each participant (27).

We also focused on the association between health outcomes in the concerned wave and JD experience before it; that is, we considered how health outcomes in wave *t* were associated with JD experience until wave *t*-1. This approach, which evaluates the presence of JD before health outcomes, is expected to mitigate simultaneity biases, which tend to overestimate the association between JD and health outcomes due to their reciprocal relationship (19, 20). Further, we examined the reverse causation from health outcomes to JD by estimating the association between health outcomes and JD one year later in the framework of mixed model analysis.

We considered three aspects of JD, evaluating it in terms of ability utilization (21, 22), workplace relationships (23, 24), and working conditions (25, 26), all of which are key determinants of JD, and combined them into a single-item measure. Regarding health outcomes, we focused on PD, poor SRH, and health-related resignation (HRR). PD measured by Kessler 6 (K6) scores (28, 29), represents mental health, SRH serves as a global measure of health status (30, 31), and HRR is a proxy for objective negative outcomes in the workplace. Given the observations in previous studies, we hypothesized that JD would be predictive of poor health outcomes in workers.

In summary, this study attempted to examine the association between JD and health outcomes and their reverse causation by applying mixed model analysis, which is expected to control for participant-specific factors that may confound the association between JD and health outcomes. The findings of this study are expected to provide new insights into the relevance of JD in occupational health to the existing literature, which consists mainly of cross-sectional or short-term prospective cohort studies.

## Methods

### Study sample

In this study, we used data obtained from a nationwide 14-wave panel survey, “The Longitudinal Survey of Middle-Aged and Older Adults,” conducted by the Japanese Ministry of Health, Labor, and Welfare (MHLW) each year from 2005 to 2018. Japan’s Statistics Law required the survey to be reviewed from statistical, legal, ethical, and other viewpoints. We obtained the survey data with the official permission of the MHLW; therefore, the current study did not require ethical approval.

The survey started with the cohort aged 50–59 years (born 1946–1955) in the first wave. A total of 34 240 individuals responded (response rate: 83.8%). The 2^nd^ to 14^th^ waves of the survey were conducted annually from 2006 to 2018; 20 677 individuals remained in the 14^th^ wave. In wave *t*, we focused on the health outcomes of participants who reported their job satisfaction in wave *t*-1. Since the questions on JD were asked of employed workers, we removed participants who had not been employed (that is, who had been unemployed, self-employed, or other) before each concerned wave. After further excluding participants with missing key health variables (PD, SRH, and HRR) in each wave, we obtained 156 823 observations of 24 056 participants (13 177 men and 10 879 women), which were used for descriptive analysis.

For regression analysis, we further removed participants who were already psychologically distressed or whose health was assessed as *poor* or *very poor* (see below for definition) one year before each concerned wave because they may have been psychologically distressed or unhealthy due to reasons other than job dissatisfaction. Consequently, we used longitudinal data from 147 830 observations of 23 498 participants (12 901 men and 10 597 women) for the regression analysis.

### Measures

#### Job dissatisfaction

The survey asked the participants to answer the question, “How do you feel about your job in terms of the following: (i) ability utilization, (ii) workplace relationships, and (iii) working conditions?” – all of which have been the key correlates of JD in preceding studies (21–26), on a five-point scale (1=satisfied, 2=somewhat satisfied, 3=average, 4=somewhat dissatisfied, and 5=dissatisfied). We calculated the sum of these scores (range: 3–15); Cronbach’s alpha varied 0.724–0.780 for each wave. We defined JD as a higher degree of overall dissatisfaction with the three job aspects; specifically, we defined it as a total score of ≥10, which accounted for 31.7% of the total respondents and roughly corresponded to the highest tertile of the total score. We then focused on how long the individuals had been continuously dissatisfied with their jobs before the concerned wave. We constructed four binary variables for JD duration: (i) ≥1 (ii) 1, (iii) 2, and (iv) ≥3 years, where (i) is a comprehensive definition of JD, including (ii)–(iv).

#### Health outcomes

We considered three health outcomes: PD, poor SRH, and HRR. We constructed K6 scores to measure the PD (28, 29). The reliability and validity of K6 has been demonstrated in a Japanese sample (32, 33). The participants were asked to answer a six-item questionnaire that included items such as “During the past 30 days, how often did you feel (a) nervous, (b) hopeless, (c) restless or fidgety, (d) so depressed that nothing could cheer you up, (e) that everything was an effort, or (f) worthless?” The questions were rated on a five-point scale (0=never to 4=all of the time). We then calculated the sum of the reported scores (range: 0–24) and defined it as the K6 score, whose Cronbach’s alpha varied 0.876–0.883 for each wave. Higher K6 scores reflect higher PD levels, and K6 scores ≥13 indicate serious mental illness in a Japanese sample, as validated by previous studies (29, 31). We constructed a binary variable for PD by assigning a value of 1 to those with K6 scores ≥13 and 0 to the others. We did not include data from respondents who did not report all six items.

Regarding SRH, the participants were asked to rate their current health condition as follows: 1 (very good), 2 (good), 3 (somewhat good), 4 (somewhat poor), 5 (poor), or 6 (very poor). SRH is correlated with morbidity and is predictive of changes in functional ability (31, 32). We constructed a binary variable for poor SRH by allocating 1 to those who chose 4–6 and 0 to the others.

We further constructed a binary variable for HRR by allocating 1 to those who answered that they stopped working for health reasons in the concerned wave and 0 to the others. This definition corresponded to a temporary leave from the workplace in most cases because the survey provided other reasons for leaving a job, such as being fired and mandatory retirement, and 95.3% of those who had resigned for health reasons resumed working in later years, allowing us to compare the results regarding sickness absence (11–13).

### Covariates

We considered a set of participant-level covariates evaluated for each wave. Specifically, we constructed binary variables for gender (female=1; male=0) and marital status (having a spouse =1; otherwise=0). For age at baseline, we constructed binary variables for each age (for instance, 1=age 50 years, 0=otherwise). Occupational status was divided into six categories (managers, regular employees, non-regular employees [such as part-time, temporary, and contract worker], other, and unemployed) and constructed binary variables for each category (for instance, 1=managers, 0=otherwise). Similarly, we constructed binary variables for each category of educational qualification (junior high school, high school, junior college, college or above, other, and unanswered) and health behavior (currently smoking, heavy alcohol consumption, and no physical activity). We also considered household spending as a proxy for household income and adjusted it for household size by dividing it by the square root of the number of household members (34). We categorized them into quartiles and constructed binary variables for each quartile. For respondents who did not answer questions about household spending, we allocated a binary variable to unanswered questions. We also included binary variables for each wave to control for wave-specific effects. We did not use occupational status in wave *t* as a covariate when estimating HRR in wave *t*, because current occupational status (especially, no job and self-employed) may be a result of HRR in many cases.

### Statistical analysis

For descriptive analysis, we compared the prevalence of each health outcome between those who were satisfied with their jobs and those who were not, and examined the extent to which the prevalence of each health outcome was correlated with JD duration.

For regression analysis, we estimated two logistic mixed models, Models 1 and 2, to predict the probability of each health outcome by JD, according to the choice of JD variables. For participant *i* in wave *t*, Model 1 is given by


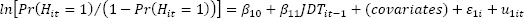


where *H* is a binary variable for each health (PD, poor SRH, or HRR), *JDT* is a binary variable for JD duration of one year or longer, ɛ_1_ represents participant-specific factors, and *u*_1_ is an error term. Meanwhile, Model 2 is given by


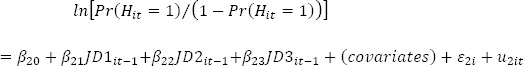


where *JD*1, *JD*2, and *JD*3 are binary variables for JD duration of one, two, and three years or longer, respectively, ɛ_2_ represents participant-specific factors, and *u*_2_ is an error term.

In both models, we focused on the association between health outcomes in the concerned wave and JD experience to mitigate simultaneity bias. We also removed the respondents with PD or poor SRH in the previous year because they may have been psychologically distressed or unhealthy due to reasons other than JD. To check the robustness of the logistic mixed model results, we estimated the linear versions of Models 1 and 2 by replacing the binary health variable, *H*, with its continuous score.

Additionally, we estimated the logistic mixed model (Model 3) to predict JD in the concerned wave. Model 3 is given by


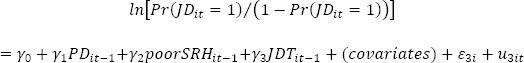


where *JD* is a binary variable for JD. *PD*, *poor SRH, and JDT* are binary variables for PD, poor SRH, and JD duration of one year or longer, respectively, all of which were evaluated in the year before each concerned wave. ɛ_3_ represents participant-specific factors, and *u*_3_ is an error term. This regression was aimed at examining the reverse causation from health to JD. To check the robustness of the estimation results, we estimated the logistic mixed model to predict JD using K6 and SRH scores instead of their binary variables. For all statistical analyses, we used the Stata software package (Release 15).

## Results

### Descriptive analysis

The proportion of participants who were dissatisfied with their jobs for ≥1 year before the concerned wave declined from 32.9% in the 2^nd^ wave to 20.9% in the 14^th^ wave. Table 1 summarizes the distribution of JD experience among the participants in the 7^th^ wave, which was around the middle of the total number of waves, and compares the prevalence of each health outcome between those who were satisfied with their jobs and those who were dissatisfied with them. As seen in this table, 27.4% of men and 21.9% of women were dissatisfied with their job for ≥1 year before the 7^th^ wave. The mean length of JD duration was 1.7 [standard deviation (SD) 1.1] years for men and 1.8 (SD 1.2) years for women. As expected, JD was associated with a higher prevalence of adverse health outcomes; for example, 3.8% of men who were dissatisfied with their jobs in the previous year experienced PD in the seventh wave, compared to 1.2% of men who were satisfied with their jobs. We also observed similar patterns in the other waves.

**Table 1:** Distribution of the study sample in terms of job dissatisfaction and health outcomes in the 7^th^ wave.

	Participants				Prevalence		
	
	N	Proportion %	Mean	SD	Psychological distress %	Poor self-rated health %	Health-related resignation %
All							
Total	13 275	100.0			2.3	15.3	1.4
Satisfied with job	9961	75.0			1.7	13.3	1.3
Dissatisfied with job							
Duration, years			1.8	1.1			
≥1	3314	25.0			4.3	21.0	1.6
1	1645	12.4			3.3	18.1	1.0
2	556	4.2			3.8	20.5	3.1
≥3	1113	8.4			5.9	25.6	1.7
Men							
All	7481	100.0			1.9	15.8	1.1
Satisfied with job	5433	72.6			1.2	13.6	1.1
Dissatisfied with job							
Duration, years			1.7	1.1			
≥1	2048	27.4			3.8	21.7	1.3
1	1051	14.0			2.9	18.0	0.8
2	333	4.5			3.3	24.0	3.0
≥3	664	8.9			5.3	26.5	1.2
Women							
All	5794	100.0			2.8	14.6	1.7
Satisfied with job	4528	78.1			2.2	13.1	1.6
Dissatisfied with job							
Duration, years			1.8	1.2			
≥1	1266	21.9			5.1	19.9	2.1
1	594	10.3		3.9	18.4	1.3
2	223	3.8			4.5	15.2	3.1
≥3	449	7.7			6.9	24.3	2.4

**Figure 1:**
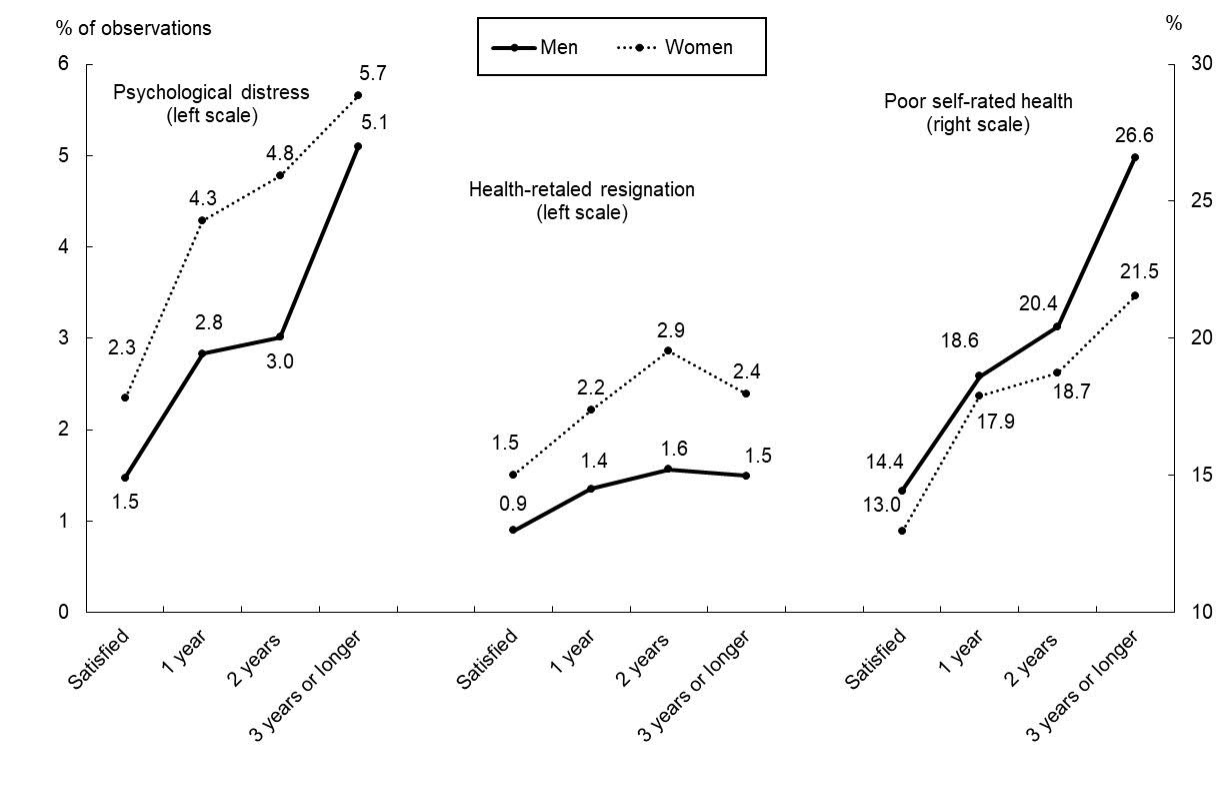
Prevalence of psychological distress by duration of job dissatisfaction. Note: ^a^ Indicated % prevelance of each health outcome among the observations with each duration of job dissastisfaction among the pooled observations (N=156 823 observations of 24 056 participants [13 177 men and 10 879 women.]

Based on pooled observations, figure 1 graphically illustrates a positive relationship between JD duration and the prevalence of each poor health outcome for both men and women. The slopes of the curves were not substantially different between genders for each health outcome, although the levels of the women’s curves were higher than those of men for PD and HRR and lower for poor SRH. These results suggest a limited interaction between JD and gender.

### Regression analysis

Table 2 presents the results of Model 1 for each health outcome. The odds ratios (OR) of reporting PD, poor SRH, and HRR in response to JD for ≥1 year were 1.96 [95% confidence interval (CI) 1.75–2.20], 1.33 (95% CI 1.26–1.40), and 1.57 (95% CI 1.40–1.73), all of which indicated a positive association between JD and poor health outcomes. We conducted a likelihood-ratio rate test to test the null hypothesis that there were no individual-level random effects. If this hypothesis is rejected, the mixed model must be applied; otherwise, we can use the pooled cross-sectional model. The test showed that the null hypothesis could be rejected (P<0.001) for all health outcomes, indicating that the mixed model was consistently preferred to the pooled cross-sectional model. In addition to these key results, the model showed that having no spouse, heavy alcohol consumption, and the lowest educational qualification (graduating from junior high school) were positively associated with poor health outcomes.

**Table 2:** Estimation results of logistic mixed models (Model 1 to predict health outcomes (N=147 830 observations of 23 498 participants. [OR=odds ratio; CI=confidence interval].

	Psychological distress		Poor self-rated health		Health-related resignation	
		
	OR ^a^	95% CI	OR ^a^	95% CI	OR ^a^	95% CI
Dissatisfied for ≥1 year	1.96	1.75–2.20	1.33	1.26–1.40	1.57	1.40–1.75
Female	1.30	1.11–1.52	0.61	0.55–0.67	1.52	1.34–1.72
Having no spouse	1.96	1.75–2.20	1.33	1.26–1.40	1.57	1.40–1.75
Health behavior						
Smoking	0.97	0.83–1.13	0.77	0.71–0.84	0.87	0.75–1.01
Heavy alcohol consumption	1.72	1.36–2.18	0.90	0.79–1.03	0.78	0.58–1.05
No exercise	1.91	1.70–2.15	1.51	1.43–1.62	0.78	0.69–0.87
Occupational status						
Manager	0.73	0.54–0.97	0.89	0.78–1.02		
Non-regular employee	0.94	0.80–1.09	0.95	0.89–1.03		
Self-employed	1.14	0.89–1.45	0.91	0.80–1.03		
Other	0.96	0.72–1.29	0.99	0.86–1.13		
No job	2.20	1.82–2.66	1.81	1.65–1.99		
Household expenditure in quartiles (ref. = 4^th^ quartile [highest]						
1^st^ (lowest)	0.98	0.83–1.16	0.83	0.77–0.91	1.71	1.45–2.02
2^nd^	0.83	0.70–0.98	0.80	0.75–0.87	1.56	1.33–1.84
3^rd^	0.94	0.81–1.09	0.90	0.84–0.97	1.28	1.08–1.50
Unanswered	1.01	0.80–1.28	0.94	0.84–1.05	1.10	0.86–1.40
Educational attainment (ref. = college or higher)						
Junior high school	1.39	1.09–1.78	2.80	2.39–3.26	1.94	1.56–2.40
High school	1.06	0.87–1.29	1.76	1.56–1.99	1.56	1.30–1.87
Junior college	1.28	0.94–1.75	1.16	0.94–1.44	1.35	1.03–1.78
Other or unanswered	1.27	0.72–2.24	2.29	1.60–3.28	1.68	1.03–2.73
Log likelihood		–9795.02		–42472.06		–8766.76
Including the interaction between job dissatisfaction and women						
Dissatisfied for ≥1 year	2.06	1.76–2.41	1.31	1.22–1.41	1.42	1.21–1.67
Female	1.35	1.13–1.61	0.61	0.55–0.67	1.43	1.24–1.65
Dissatisfied for ≥1 year × female	0.90	0.71–1.13	1.03	0.92–1.15	1.21	0.96–1.51
Log likelihood		–9794.56		–42471.94		–8765.41

a Odds ratio for reporting each health outcome. Further adjustments were made for age at baseline and for the waves.

The bottom of table 2 presents the key estimation results obtained after including the interaction term between JD and being female. The OR of the interaction term was not significantly different from that in all models, while the OR of the interaction term in logistic regression models could not be interpreted in a straightforward manner (35). Thus, we present the regression results for the entire sample, rather than separately for men and women in what follows.

Table 3 summarizes the key results of Models 1 and 2, adjusted for covariates, and compares the results between the logistic and linear models. These three findings are noteworthy. First, JD was positively associated with poor health outcomes in all model specifications. Second, a longer JD duration was associated with poorer health outcomes in most model specifications, a result consistent with the observations in table 1 and Figure 1. For instance, as JD duration increased from 1 to ≥3 years, the OR of reporting PD increased from 1.67 (95% CI 1.42–1.96) to 2.49 (95% CI 2.09–2.96) in the mixed model. Third, the results obtained using the linear models were consistent with those obtained from the logistic results.

**Table 3:** Estimation results of mixed models (Models 1 and 2) to predict health outcomes (N = 147 830 observations of 23 498 participants). [OR=odds ratio; CI=confidence interval]

	Psychological distress		Poor self-rated health		Health-related resignation	
		
	OR ^a^	95% CI	OR ^a^	95% CI	OR ^a^	95% CI
Logistic mixed models						
Model 1	1.96	1.75–2.20	1.33	1.26–1.40	1.57	1.40–1.75
≥1 year						
Model 2 (years)						
1	1.67	1.42–1.96	1.19	1.10–1.28	1.34	1.14–1.57
2	1.76	1.40–2.22	1.30	1.17–1.45	1.71	1.38–2.13
≥3	2.49	2.09–2.96	1.61	1.46–1.77	1.61	1.35–2.91
			
	K6 score ^b^(range: 0–24)		Self-rated health score ^c^(range: 1–6)		Health-related resignation (range: 0–1)	
		
	Coefficent	95% CI	Coefficent	95% CI	Coefficent	95% CI
		
Linear mixed models						
Model 1						
≥1	0.27	0.24–0.31	0.05	0.04–0.06	0.006	0.004–0.007
Model 2 (years)						
1	0.16	0.12–0.21	0.03	0.02–0.04	0.004	0.002–0.006
2	0.27	0.21–0.34	0.05	0.04–0.07	0.007	0.004–0.011
≥3	0.45	0.39–0.52	0.10	0.08–0.11	0.006	0.004–0.008

a Odds ratio for reporting each health outcome Adjusted for covariates (gender, marital status, health behavior, occupational status, household expenditure, educational attainment, age at baseline, and waves).

b The higher, the more distressed.

c The higher, the poorer health.

Table 4 presents the results of Model 3, which explains a binary variable for JD in the concerned wave by one-year lagged variables for PD, poor SRH, and JD. The table showed that JD was positively associated with both PD and poor SRH in the previous year with an OR of 1.50 (95% CI 1.35–1.67) and 1.37 (95% CI 1.30–1.43), respectively. The bottom panel of the table confirms that replacing binary variables for health with the continuous variables obtained results that were consistent with those in the binary model.

**Table 4:** Estimation results of logistic regression models (Model 3) to predict job dissatisfaction (N=136 998 observations of 22 058 participants). [OR=odds ratio; CI=confidence interval].

	OR ^a^	95% CI
Using binary variables of health		
Psychological distress (lagged)	1.50	1.35–1.67
Poor self-rated health (lagged)	1.37	1.30–1.43
Job dissatisfaction (lagged)	3.28	3.16–3.41
Using continuous variables of health		
K6 score ^b^(lagged, range: 0–24)	1.18	1.16–1.20
Self-rated health score ^c^ (lagged; range: 1–6)	1.13	1.11–1.15
Job dissatisfaction (lagged)	3.23	3.11–3.36

a Odds ratio for reporting job dissatisfaction (for K6 and self-rated health scores, in response to their 1-standard deviation increases). Adjusted for covariates (gender, marital status, health behavior, occupational status, household expenditure, educational attainment, age at baseline, and waves).

b The higher, the more distressed.

c The higher, the poorer health.

## Discussion

In this study, we examined the association between JD and health outcomes among middle-aged workers, controlling for individual attributes. To take full advantage of repeated measures for each participant over time, we conducted a mixed model analysis of the 14-wave longitudinal data of middle-aged workers. As hypothesized, the results indicated that JD is a key predictor of adverse health outcomes in workers.

These results were generally consistent with those obtained in previous studies (3–13), most of which were based on cross-sectional or prospective cohort studies. In addition, the observed impact on HRR was in line with that reported in previous studies on the impact of JD on negative outcomes in the workplace (11–16). We confirmed the validity of the conventional view that JD is an important determinant of occupational health, even when controlling for participant-specific attributes.

Second, we observed that a longer JD duration moderately added to the risks of each poor health outcome, as shown in both the descriptive and mixed model analyses. While studies have shown that JD tended to discourage workers from staying in the workplace (11–16), the results suggest that a prolonged JD duration was associated with an average deterioration in health outcomes.

Third, behind this accumulating effect of JD duration, there may be cumulative causation between JD and health outcomes. As seen in table 4, JD was not only affected by JD duration until the previous year but also by the previous year’s PD and poor SRH, which had been affected by JD duration beforehand, as suggested by the results in table 3. The existence of the latter route suggests that PD and poor SRH may mediate the impact of previous PD experience on current PD. This mediation mechanism is likely to enhance JD’s self-sustainability and thus make JD adversely affect health over time.

This study focused on middle-aged workers, who were more exposed to the onset of health problems compared to other age groups (36, 37). Hence, the observations in this study may help assess the potential magnitude of the association between JD and health in all age groups. However, many studies have shown that age can be a moderator in the association between work characteristics and occupational well-being indicators (38); for instance, JD is known to be more sensitive to job instability among younger than older workers (39). Hence, it is reasonable to suspect that the association between JD and health outcomes may differ across age groups, suggesting the need to expand the analysis to cover workers of all ages.

This study has several limitations. First, we ignored the possibility that JD may have various aspects and that each may affect health outcomes independently and differently because we combined three aspects of JD – dissatisfaction with ability utilization, workplace relationships, and working conditions – into a single-item measure. Previous observations that job dissatisfaction is substantially affected by psychosocial work factors, such as support from superiors and colleagues (40, 41), suggest a closer association of dissatisfaction with workplace relationships. Therefore, the relative effect of each JD aspect on PD and their relationships should be examined in future research.

Second, the association between job satisfaction and health outcomes is likely to be confounded by job status, especially in full-time and part-time jobs. Low job satisfaction and poor health status are more common among part-time than full-time workers (42), and the confounding effects of job status may be affected by socio-institutional factors related to equal treatment between full- and part-time workers. The analysis of these effects remains a topic for future research.

Third, this study ignored potential biases due to attrition. The proportion of participants who were dissatisfied with their job declined over the waves, suggesting the possibility that the observed association between JD and health outcomes may have been underestimated. Mixed models in this study focused on the short-term association between JD and health, but the analysis of the long-term impact of JD on health should control for potential attrition biases considering the possibility that unhealthy participants were more likely to have dropped out of the survey.

### Concluding remarks

Despite these limitations, we can conclude that JD is modestly associated with poor health among middle-aged workers. Policymakers and managers should regularly monitor employees’ job dissatisfaction and make efforts to improve their work environments to enhance their occupational health.

## References

[1] Arnold AE, Coffeng JK, Boot CR, van der Beek AJ, van Tulder MW, Nieboer D (2016). The relationship between job satisfaction and productivity-related costs:a longitudinal analysis. J Occup Environ Med.

[2] Faragher EB, Cass M, Cooper CL (2005). The relationship between job satisfaction and health:a meta-analysis. Occup Environ Med.

[3] Stansfeld S, Candy B (2006). Psychosocial work environment and mental health--a meta-analytic review. Scand J Work Environ Health.

[4] Nagai M, Tsuchiya KJ, Toulopoulou T, Takei N (2007). Poor mental health associated with job dissatisfaction among school teachers in Japan. J Occup Health.

[5] Nakata A (2017). Long working hours, job satisfaction, and depressive symptoms:a community-based cross-sectional study among Japanese employees in small- and medium-scale businesses. Oncotarget.

[6] Tatsuse T, Sekine M (2013). Job dissatisfaction as a contributor to stress-related mental health problems among Japanese civil servants. Ind Health.

[7] Tatsuse T, Sekine M, Yamada M (2019). The contributions made by job satisfaction and psychosocial stress to the development and persistence of depressive symptoms:a 1-year prospective study. J Occup Environ Med.

[8] D'Angelo S, Coggon D, Harris EC, Linaker C, Sayer AA, Gale CR (2016). Job dissatisfaction and the older worker:baseline findings from the Health and Employment After Fifty study. Occup Environ Med.

[9] Fischer JA, Sousa-Poza A (2009). Does job satisfaction improve the health of workers?New evidence using panel data and objective measures of health. Health Econ.

[10] Navarro-Abal Y, Sáenz-de la Torre LC, Gómez-Salgado J, Climent-Rodríguez JA (2018). Job satisfaction and perceived health in Spanish construction workers during the economic crisis. Int J Environ Res Public Health.

[11] Böckerman P, Ilmakunnas P (2008). Interaction of working conditions, job satisfaction, and sickness absences:evidence from a representative sample of employees. Soc Sci Med.

[12] Hoogendoorn WE, Bongers PM, de Vet HC, Ariëns GA, van Mechelen W, Bouter LM (2002). High physical work load and low job satisfaction increase the risk of sickness absence due to low back pain:results of a prospective cohort study. Occup Environ Med.

[13] Nakata A, Takahashi M, Irie M, Ray T, Swanson NG (2011). Job satisfaction, common cold, and sickness absence among white-collar employees:a cross-sectional survey. Ind Health.

[14] D'Angelo S, Syddall H, Ntani G, Harris EC, Linaker C, Cooper C (2021). How does job dissatisfaction interact with self-rated health in determining the risk of health-related job loss?Prospective findings from the Health and Employment After Fifty (HEAF) study. Occup Environ Med.

[15] Albertsen K, Lund T, Christensen KB, Kristensen TS, Villadsen E (2007). Predictors of disability pension over a 10-year period for men and women. Scand J Public Health.

[16] Labriola M, Feveile H, Christensen KB, Bültmann U, Lund T (2009). The impact of job satisfaction on the risk of disability pension. A 15-year prospective study. Scand J Public Health.

[17] Rukh G, Dang J, Olivo G, Ciuculete DM, Rask-Andersen M, Schiöth HB (2020). Personality, lifestyle and job satisfaction:causal association between neuroticism and job satisfaction using Mendelian randomisation in the UK biobank cohort. Transl Psychiatry.

[18] Törnroos M, Jokela M, Hakulinen C (2019). The relationship between personality and job satisfaction across occupations. Pers Individ Dif.

[19] Antonakis J, Bendahan S, Jacquart P, Lalive R (2010). On making causal claims:a review and recommendations. Leadersh Q.

[20] Gennetian LA, Magnuson K, Morris PA (2008). From statistical associations to causation:what developmentalists can learn from instrumental variables techniques coupled with experimental data. Dev Psychol.

[21] Fujishiro K, Heaney CA (2017). “Doing what I do best”:the association between skill utilization and employee health with healthy behavior as a mediator. Soc Sci Med.

[22] Morrison D, Cordery J, Girardi A, Payne R (2005). Job design, opportunities for skill utilization, and intrinsic job satisfaction. Job design, opportunities for skill utilization, and intrinsic job satisfaction. Eur J Work Organ Psychol.

[23] van Beek AP, Wagner C, Spreeuwenberg PP, Frijters DH, Ribbe MW, Groenewegen PP (2011). Communication, advice exchange and job satisfaction of nursing staff:a social network analyses of 35 long-term care units. BMC Health Serv Res.

[24] Yuan CT, Lai AY, Benishek LE, Marsteller JA, Mahabare D, Kharrazi H (2021). A double-edged sword:the effects of social network ties on job satisfaction in primary care organizations. Health Care Manage Rev.

[25] Böckerman P, Ilmakunnas P (2008). Interaction of working conditions, job satisfaction, and sickness absences:evidence from a representative sample of employees. Soc Sci Med.

[26] Wada K, Arimatsu M, Higashi T, Yoshikawa T, Oda S, Taniguchi H (2009). Physician job satisfaction and working conditions in Japan. J Occup Health.

[27] Detry MA, Ma Y (2016). Analyzing repeated measurements using mixed models. JAMA.

[28] Kessler RC, Andrews G, Colpe LJ, Hiripi E, Mroczek DK, Normand SL (2002). Short screening scales to monitor population prevalences and trends in non-specific psychological distress. Psychol Med.

[29] Kessler RC, Green JG, Gruber MJ, Sampson NA, Bromet E, Cuitan M (2010). Screening for serious mental illness in the general population with the K6 screening scale:results from the WHO World Mental Health (WMH) survey initiative. Int J Methods Psychiatr Res.

[30] Idler EL, Benyamini Y (1997). Self-rated health and mortality:a review of twenty-seven community studies. J Health Soc Behav.

[31] Wu S, Wang R, Zhao Y, Ma X, Wu M, Yan X (2013). The relationship between self-rated health and objective health status:a population-based study. BMC Public Health.

[32] Furukawa TA, Kawakami N, Saitoh M, Ono Y, Nakane Y, Nakamura Y (2008). The performance of the Japanese version of the K6 and K10 in the World Mental Health Survey Japan. Int J Methods Psychiatr Res.

[33] Sakurai K, Nishi A, Kondo K, Yanagida K, Kawakami N (2011). Screening performance of K6/K10 and other screening instruments for mood and anxiety disorders in Japan. Psychiatry Clin Neurosci.

[34] Organisation for Economic Co-operation and Development (2015). In it together:why less inequality benefits all.

[35] Chen JJ (2003). Communicating complex information:the interpretation of statistical interaction in multiple logistic regression analysis. Am J Public Health.

[36] Countdown NC;NCD Countdown 2030 collaborators. NCD Countdown 2030:worldwide trends in non-communicable disease mortality and progress towards Sustainable Development Goal target 3.4 (2018). Lancet.

[37] Ministry of Health. Labour and Welfare c2020 [Internet] (2019). The National Health and Nutrition Survey in Japan.

[38] Zacher H, Schmitt A (2016). Work characteristics and occupational well-being:the role of age. Front Psychol.

[39] Mauno S, Ruokolainen M, Kinnunen U (2013). Does aging make employees more resilient to job stress?Age as a moderator in the job stressor-well-being relationship in three Finnish occupational samples. Aging Ment Health.

[40] Andersen LL, Fishwick D, Robinson E, Wiezer NM, Mockałło Z, Grosjean V (2017). Job satisfaction is more than a fruit basket, health checks and free exercise:cross-sectional study among 10,000 wage earners. Scand J Public Health.

[41] Inoue A, Tsutsumi A, Kachi Y, Eguchi H, Shimazu A, Kawakami N (2020). Psychosocial work environment explains the association of job dissatisfaction with long-term sickness absence:a one-year prospect study of Japanese employees. J Epidemiol.

[42] Bartoll X, Cortès I, Artazcoz L (2014). Full- and part-time work:gender and welfare-type differences in European working conditions, job satisfaction, health status, and psychosocial issues. Scand J Work Environ Health.

